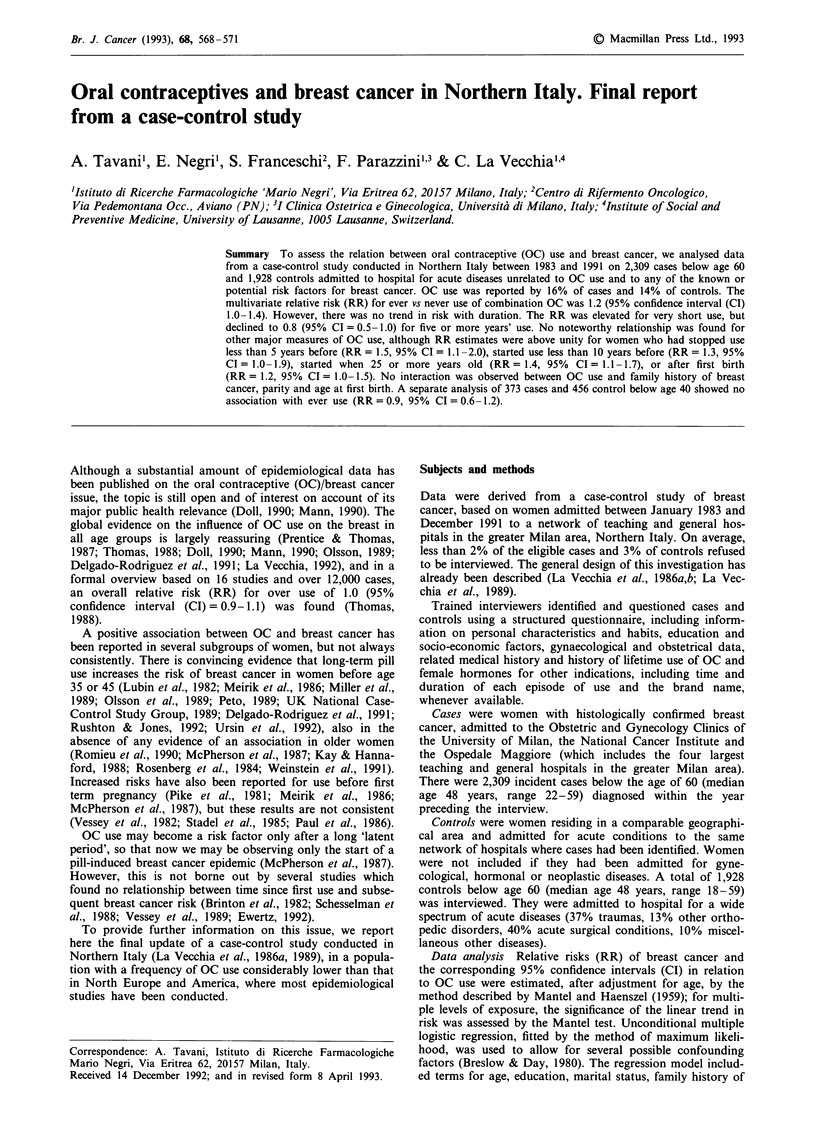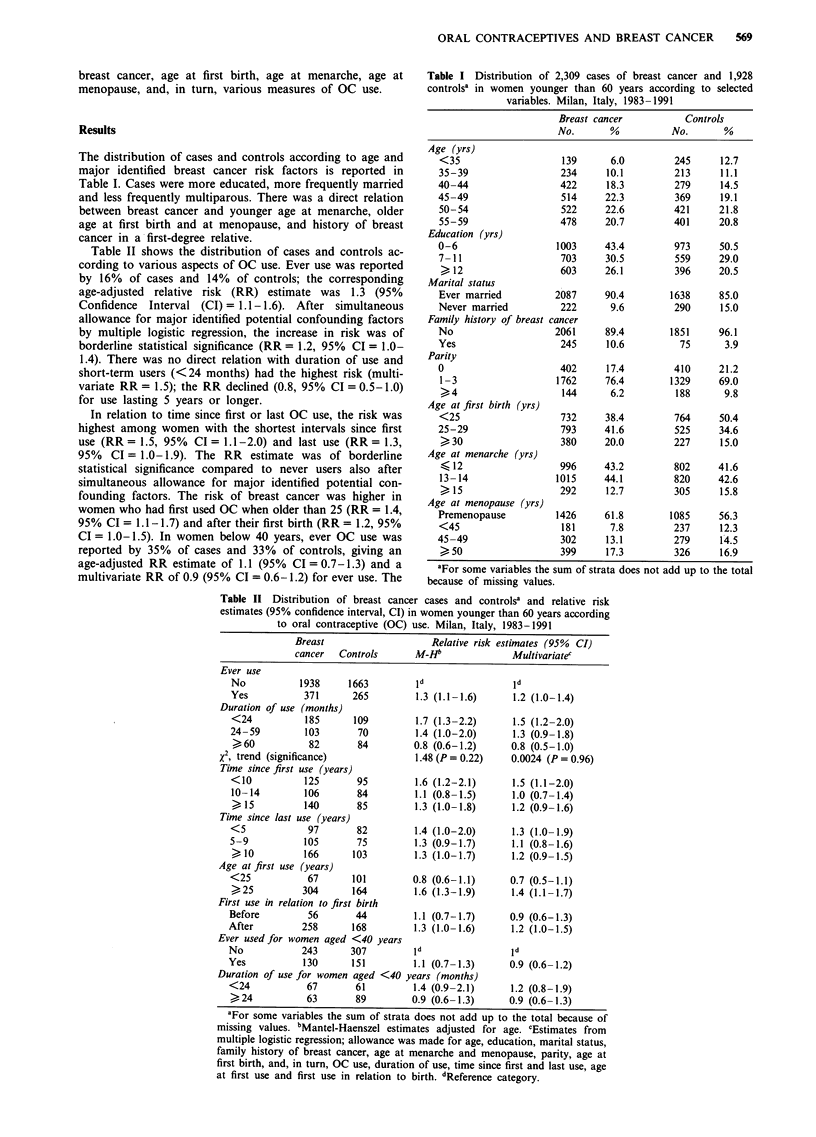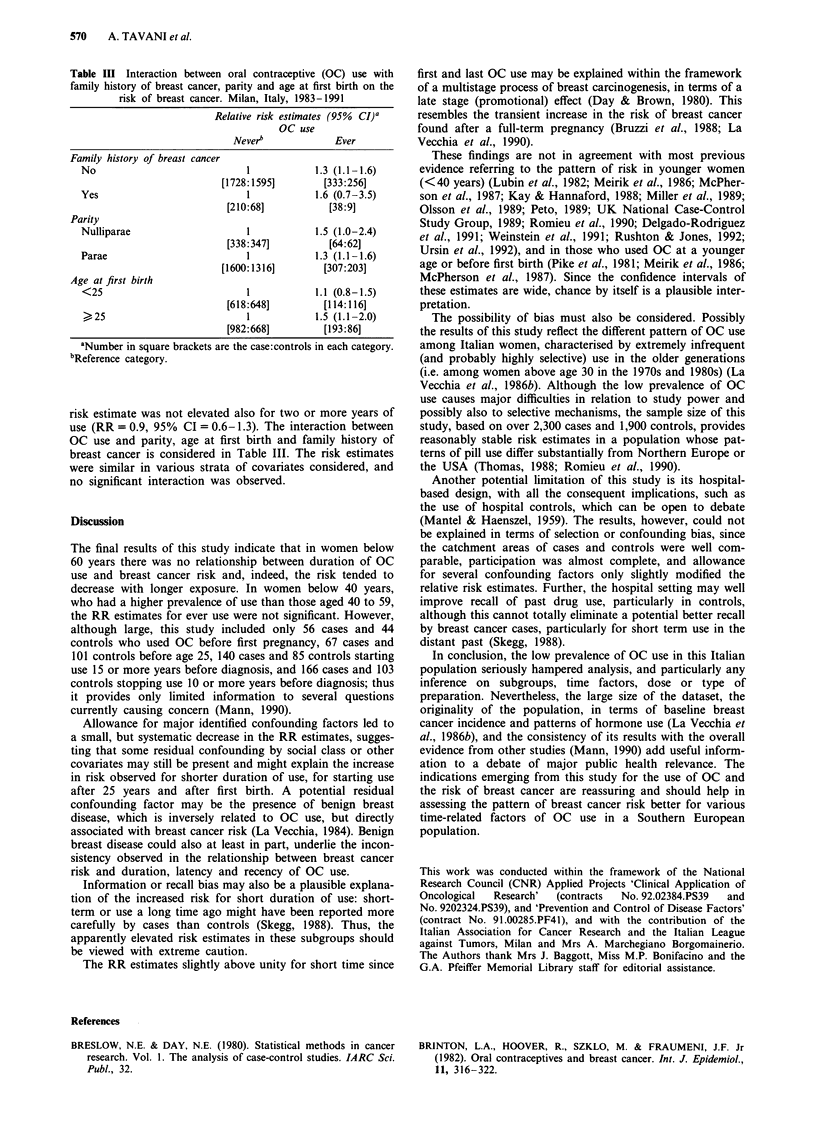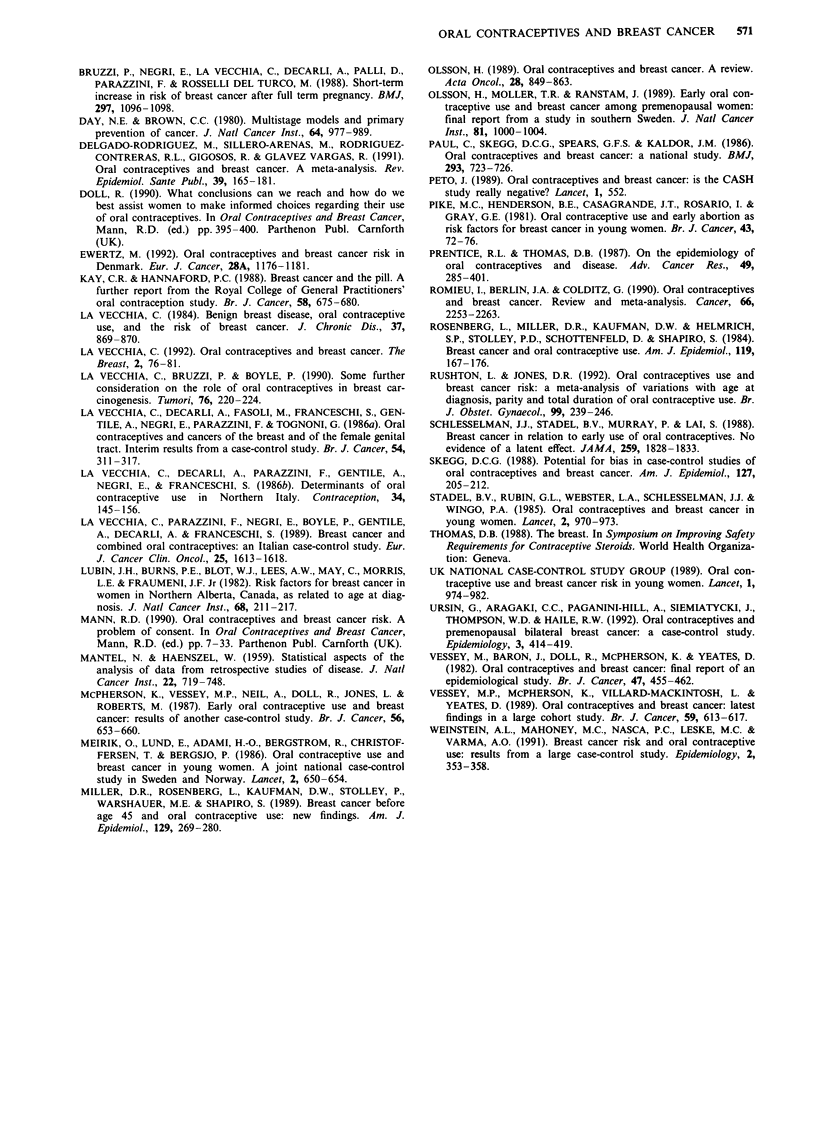# Oral contraceptives and breast cancer in northern Italy. Final report from a case-control study.

**DOI:** 10.1038/bjc.1993.387

**Published:** 1993-09

**Authors:** A. Tavani, E. Negri, S. Franceschi, F. Parazzini, C. La Vecchia

**Affiliations:** Istituto di Ricerche Farmacologiche Mario Negri, Milano, Italy.

## Abstract

To assess the relation between oral contraceptive (OC) use and breast cancer, we analysed data from a case-control study conducted in Northern Italy between 1983 and 1991 on 2,309 cases below age 60 and 1,928 controls admitted to hospital for acute diseases unrelated to OC use and to any of the known or potential risk factors for breast cancer. OC use was reported by 16% of cases and 14% of controls. The multivariate relative risk (RR) for ever vs never use of combination OC was 1.2 (95% confidence interval (CI) 1.0-1.4). However, there was no trend in risk with duration. The RR was elevated for very short use, but declined to 0.8 (95% CI = 0.5-1.0) for five or more years' use. No noteworthy relationship was found for other major measures of OC use, although RR estimates were above unity for women who had stopped use less than 5 years before (RR = 1.5, 95% CI = 1.1-2.0), started use less than 10 years before (RR = 1.3, 95% CI = 1.0-1.9), started when 25 or more years old (RR = 1.4, 95% CI = 1.1-1.7), or after first birth (RR = 1.2, 95% CI = 1.0-1.5). No interaction was observed between OC use and family history of breast cancer, parity and age at first birth. A separate analysis of 373 cases and 456 control below age 40 showed no association with ever use (RR = 0.9, 95% CI = 0.6-1.2).


Br. J. Cancer (1993), 68, 568-571 © Macmillan Press Ltd., 1993

### Oral contraceptives and breast cancer in Northern Italy. Final report from a case-control study

A. Tavani[1], E. Negri[1], S. Franceschi[2], F. Parazzini[1,3] & C. La Vecchia[1,4]

[1]*Istituto di Ricerche Farmacologiche 'Mario Negri', Via Eritrea 62, 20157 Milano, Italy;* [2]*Centro di Rifermento Oncologico, Via Pedemontana Occ., Aviano (PN);* [3]*I Clinica Ostetrica e Ginecologica, Università di Milano, Italy;* [4]*Institute of Social and Preventive Medicine, University of Lausanne, 1005 Lausanne, Switzerland.*

**Summary** To assess the relation between oral contraceptive (OC) use and breast cancer, we analysed data from a case-control study conducted in Northern Italy between 1983 and 1991 on 2,309 cases below age 60 and 1,928 controls admitted to hospital for acute diseases unrelated to OC use and to any of the known or potential risk factors for breast cancer. OC use was reported by 16% of cases and 14% of controls. The multivariate relative risk (RR) for ever *vs* never use of combination OC was 1.2 (95% confidence interval (CI) 1.0-1.4). However, there was no trend in risk with duration. The RR was elevated for very short use, but declined to 0.8 (95% CI = 0.5-1.0) for five or more years' use. No noteworthy relationship was found for other major measures of OC use, although RR estimates were above unity for women who had stopped use less than 5 years before (RR = 1.5, 95% CI = 1.1-2.0), started use less than 10 years before (RR = 1.3, 95% CI = 1.0-1.9), started when 25 or more years old (RR = 1.4, 95% CI = 1.1-1.7), or after first birth (RR = 1.2, 95% CI = 1.0-1.5). No interaction was observed between OC use and family history of breast cancer, parity and age at first birth. A separate analysis of 373 cases and 456 control below age 40 showed no association with ever use (RR = 0.9, 95% CI = 0.6-1.2).

Although a substantial amount of epidemiological data has been published on the oral contraceptive (OC)/breast cancer issue, the topic is still open and of interest on account of its major public health relevance (Doll, 1990; Mann, 1990). The global evidence on the influence of OC use on the breast in all age groups is largely reassuring (Prentice & Thomas, 1987; Thomas, 1988; Doll, 1990; Mann, 1990; Olsson, 1989; Delgado-Rodriguez *et al.*, 1991; La Vecchia, 1992), and in a formal overview based on 16 studies and over 12,000 cases, an overall relative risk (RR) for over use of 1.0 (95% confidence interval (CI) = 0.9-1.1) was found (Thomas, 1988).

A positive association between OC and breast cancer has been reported in several subgroups of women, but not always consistently. There is convincing evidence that long-term pill use increases the risk of breast cancer in women before age 35 or 45 (Lubin *et al.*, 1982; Meirik *et al.*, 1986; Miller *et al.*, 1989; Olsson *et al.*, 1989; Peto, 1989; UK National Case-Control Study Group, 1989; Delgado-Rodriguez *et al.*, 1991; Rushton & Jones, 1992; Ursin *et al.*, 1992), also in the absence of any evidence of an association in older women (Romieu *et al.*, 1990; McPherson *et al.*, 1987; Kay & Hannaford, 1988; Rosenberg *et al.*, 1984; Weinstein *et al.*, 1991). Increased risks have also been reported for use before first term pregnancy (Pike *et al.*, 1981; Meirik *et al.*, 1986; McPherson *et al.*, 1987), but these results are not consistent (Vessey *et al.*, 1982; Stadel *et al.*, 1985; Paul *et al.*, 1986).

OC use may become a risk factor only after a long 'latent period', so that now we may be observing only the start of a pill-induced breast cancer epidemic (McPherson *et al.*, 1987). However, this is not borne out by several studies which found no relationship between time since first use and subsequent breast cancer risk (Brinton *et al.*, 1982; Schesselman *et al.*, 1988; Vessey *et al.*, 1989; Ewertz, 1992).

To provide further information on this issue, we report here the final update of a case-control study conducted in Northern Italy (La Vecchia *et al.*, 1986*a*, 1989), in a population with a frequency of OC use considerably lower than that in North Europe and America, where most epidemiological studies have been conducted.

Correspondence: A. Tavani, Istituto di Ricerche Farmacologiche Mario Negri, Via Eritrea 62, 20157 Milan, Italy.
Received 14 December 1992; and in revised form 8 April 1993.

### Subjects and methods

Data were derived from a case-control study of breast cancer, based on women admitted between January 1983 and December 1991 to a network of teaching and general hospitals in the greater Milan area, Northern Italy. On average, less than 2% of the eligible cases and 3% of controls refused to be interviewed. The general design of this investigation has already been described (La Vecchia *et al.*, 1986*a*,*b*; La Vecchia *et al.*, 1989).

Trained interviewers identified and questioned cases and controls using a structured questionnaire, including information on personal characteristics and habits, education and socio-economic factors, gynaecological and obstetrical data, related medical history and history of lifetime use of OC and female hormones for other indications, including time and duration of each episode of use and the brand name, whenever available.

*Cases* were women with histologically confirmed breast cancer, admitted to the Obstetric and Gynecology Clinics of the University of Milan, the National Cancer Institute and the Ospedale Maggiore (which includes the four largest teaching and general hospitals in the greater Milan area). There were 2,309 incident cases below the age of 60 (median age 48 years, range 22-59) diagnosed within the year preceding the interview.

*Controls* were women residing in a comparable geographical area and admitted for acute conditions to the same network of hospitals where cases had been identified. Women were not included if they had been admitted for gynecological, hormonal or neoplastic diseases. A total of 1,928 controls below age 60 (median age 48 years, range 18-59) was interviewed. They were admitted to hospital for a wide spectrum of acute diseases (37% traumas, 13% other orthopedic disorders, 40% acute surgical conditions, 10% miscellaneous other diseases).

*Data analysis* Relative risks (RR) of breast cancer and the corresponding 95% confidence intervals (CI) in relation to OC use were estimated, after adjustment for age, by the method described by Mantel and Haenszel (1959); for multiple levels of exposure, the significance of the linear trend in risk was assessed by the Mantel test. Unconditional multiple logistic regression, fitted by the method of maximum likelihood, was used to allow for several possible confounding factors (Breslow & Day, 1980). The regression model included terms for age, education, marital status, family history of breast cancer, age at first birth, age at menarche, age at menopause, and, in turn, various measures of OC use.

### Results

The distribution of cases and controls according to age and major identified breast cancer risk factors is reported in Table I. Cases were more educated, more frequently married and less frequently multiparous. There was a direct relation between breast cancer and younger age at menarche, older age at first birth and at menopause, and history of breast cancer in a first-degree relative.

Table II shows the distribution of cases and controls according to various aspects of OC use. Ever use was reported by 16% of cases and 14% of controls; the corresponding age-adjusted relative risk (RR) estimate was 1.3 (95% Confidence Interval (CI) = 1.1–1.6). After simultaneous allowance for major identified potential confounding factors by multiple logistic regression, the increase in risk was of borderline statistical significance (RR = 1.2, 95% CI = 1.0–1.4). There was no direct relation with duration of use and short-term users (<24 months) had the highest risk (multivariate RR = 1.5); the RR declined (0.8, 95% CI = 0.5–1.0) for use lasting 5 years or longer.

In relation to time since first or last OC use, the risk was highest among women with the shortest intervals since first use (RR = 1.5, 95% CI = 1.1–2.0) and last use (RR = 1.3, 95% CI = 1.0–1.9). The RR estimate was of borderline statistical significance compared to never users also after simultaneous allowance for major identified potential confounding factors. The risk of breast cancer was higher in women who had first used OC when older than 25 (RR = 1.4, 95% CI = 1.1–1.7) and after their first birth (RR = 1.2, 95% CI = 1.0–1.5). In women below 40 years, ever OC use was reported by 35% of cases and 33% of controls, giving an age-adjusted RR estimate of 1.1 (95% CI = 0.7–1.3) and a multivariate RR of 0.9 (95% CI = 0.6–1.2) for ever use. The

**Table I** Distribution of 2,309 cases of breast cancer and 1,928 controls[a] in women younger than 60 years according to selected variables. Milan, Italy, 1983–1991

| | *Breast cancer* | | *Controls* | |
|---|---|---|---|---|
| | *No.* | *%* | *No.* | *%* |
| *Age (yrs)* | | | | |
| <35 | 139 | 6.0 | 245 | 12.7 |
| 35–39 | 234 | 10.1 | 213 | 11.1 |
| 40–44 | 422 | 18.3 | 279 | 14.5 |
| 45–49 | 514 | 22.3 | 369 | 19.1 |
| 50–54 | 522 | 22.6 | 421 | 21.8 |
| 55–59 | 478 | 20.7 | 401 | 20.8 |
| *Education (yrs)* | | | | |
| 0–6 | 1003 | 43.4 | 973 | 50.5 |
| 7–11 | 703 | 30.5 | 559 | 29.0 |
| ⩾12 | 603 | 26.1 | 396 | 20.5 |
| *Marital status* | | | | |
| Ever married | 2087 | 90.4 | 1638 | 85.0 |
| Never married | 222 | 9.6 | 290 | 15.0 |
| *Family history of breast cancer* | | | | |
| No | 2061 | 89.4 | 1851 | 96.1 |
| Yes | 245 | 10.6 | 75 | 3.9 |
| *Parity* | | | | |
| 0 | 402 | 17.4 | 410 | 21.2 |
| 1–3 | 1762 | 76.4 | 1329 | 69.0 |
| ⩾4 | 144 | 6.2 | 188 | 9.8 |
| *Age at first birth (yrs)* | | | | |
| <25 | 732 | 38.4 | 764 | 50.4 |
| 25–29 | 793 | 41.6 | 525 | 34.6 |
| ⩾30 | 380 | 20.0 | 227 | 15.0 |
| *Age at menarche (yrs)* | | | | |
| ⩽12 | 996 | 43.2 | 802 | 41.6 |
| 13–14 | 1015 | 44.1 | 820 | 42.6 |
| ⩾15 | 292 | 12.7 | 305 | 15.8 |
| *Age at menopause (yrs)* | | | | |
| Premenopause | 1426 | 61.8 | 1085 | 56.3 |
| <45 | 181 | 7.8 | 237 | 12.3 |
| 45–49 | 302 | 13.1 | 279 | 14.5 |
| ⩾50 | 399 | 17.3 | 326 | 16.9 |

[a]For some variables the sum of strata does not add up to the total because of missing values.

**Table II** Distribution of breast cancer cases and controls[a] and relative risk estimates (95% confidence interval, CI) in women younger than 60 years according to oral contraceptive (OC) use. Milan, Italy, 1983–1991

| | Breast cancer | Controls | *Relative risk estimates (95% CI)* M-H[b] | Multivariate[c] |
|---|---|---|---|---|
| *Ever use* | | | | |
| No | 1938 | 1663 | 1[d] | 1[d] |
| Yes | 371 | 265 | 1.3 (1.1–1.6) | 1.2 (1.0–1.4) |
| *Duration of use (months)* | | | | |
| <24 | 185 | 109 | 1.7 (1.3–2.2) | 1.5 (1.2–2.0) |
| 24–59 | 103 | 70 | 1.4 (1.0–2.0) | 1.3 (0.9–1.8) |
| ⩾60 | 82 | 84 | 0.8 (0.6–1.2) | 0.8 (0.5–1.0) |
| $\chi^2$, trend (significance) | | | 1.48 ($P$ = 0.22) | 0.0024 ($P$ = 0.96) |
| *Time since first use (years)* | | | | |
| <10 | 125 | 95 | 1.6 (1.2–2.1) | 1.5 (1.1–2.0) |
| 10–14 | 106 | 84 | 1.1 (0.8–1.5) | 1.0 (0.7–1.4) |
| ⩾15 | 140 | 85 | 1.3 (1.0–1.8) | 1.2 (0.9–1.6) |
| *Time since last use (years)* | | | | |
| <5 | 97 | 82 | 1.4 (1.0–2.0) | 1.3 (1.0–1.9) |
| 5–9 | 105 | 75 | 1.3 (0.9–1.7) | 1.1 (0.8–1.6) |
| ⩾10 | 166 | 103 | 1.3 (1.0–1.7) | 1.2 (0.9–1.5) |
| *Age at first use (years)* | | | | |
| <25 | 67 | 101 | 0.8 (0.6–1.1) | 0.7 (0.5–1.1) |
| ⩾25 | 304 | 164 | 1.6 (1.3–1.9) | 1.4 (1.1–1.7) |
| *First use in relation to first birth* | | | | |
| Before | 56 | 44 | 1.1 (0.7–1.7) | 0.9 (0.6–1.3) |
| After | 258 | 168 | 1.3 (1.0–1.6) | 1.2 (1.0–1.5) |
| *Ever used for women aged <40 years* | | | | |
| No | 243 | 307 | 1[d] | 1[d] |
| Yes | 130 | 151 | 1.1 (0.7–1.3) | 0.9 (0.6–1.2) |
| *Duration of use for women aged <40 years (months)* | | | | |
| <24 | 67 | 61 | 1.4 (0.9–2.1) | 1.2 (0.8–1.9) |
| ⩾24 | 63 | 89 | 0.9 (0.6–1.3) | 0.9 (0.6–1.3) |

[a]For some variables the sum of strata does not add up to the total because of missing values. [b]Mantel-Haenszel estimates adjusted for age. [c]Estimates from multiple logistic regression; allowance was made for age, education, marital status, family history of breast cancer, age at menarche and menopause, parity, age at first birth, and, in turn, OC use, duration of use, time since first and last use, age at first use and first use in relation to birth. [d]Reference category.

**Table III** Interaction between oral contraceptive (OC) use with family history of breast cancer, parity and age at first birth on the risk of breast cancer. Milan, Italy, 1983–1991

| | *Relative risk estimates (95% CI)[a] OC use* | |
|---|---|---|
| | *Never[b]* | *Ever* |
| *Family history of breast cancer* | | |
| No | 1 | 1.3 (1.1–1.6) |
| | [1728:1595] | [333:256] |
| Yes | 1 | 1.6 (0.7–3.5) |
| | [210:68] | [38:9] |
| *Parity* | | |
| Nulliparae | 1 | 1.5 (1.0–2.4) |
| | [338:347] | [64:62] |
| Parae | 1 | 1.3 (1.1–1.6) |
| | [1600:1316] | [307:203] |
| *Age at first birth* | | |
| <25 | 1 | 1.1 (0.8–1.5) |
| | [618:648] | [114:116] |
| ⩾25 | 1 | 1.5 (1.1–2.0) |
| | [982:668] | [193:86] |

[a]Number in square brackets are the case:controls in each category. [b]Reference category.

risk estimate was not elevated also for two or more years of use (RR = 0.9, 95% CI = 0.6–1.3). The interaction between OC use and parity, age at first birth and family history of breast cancer is considered in Table III. The risk estimates were similar in various strata of covariates considered, and no significant interaction was observed.

### Discussion

The final results of this study indicate that in women below 60 years there was no relationship between duration of OC use and breast cancer risk and, indeed, the risk tended to decrease with longer exposure. In women below 40 years, who had a higher prevalence of use than those aged 40 to 59, the RR estimates for ever use were not significant. However, although large, this study included only 56 cases and 44 controls who used OC before first pregnancy, 67 cases and 101 controls before age 25, 140 cases and 85 controls starting use 15 or more years before diagnosis, and 166 cases and 103 controls stopping use 10 or more years before diagnosis; thus it provides only limited information to several questions currently causing concern (Mann, 1990).

Allowance for major identified confounding factors led to a small, but systematic decrease in the RR estimates, suggesting that some residual confounding by social class or other covariates may still be present and might explain the increase in risk observed for shorter duration of use, for starting use after 25 years and after first birth. A potential residual confounding factor may be the presence of benign breast disease, which is inversely related to OC use, but directly associated with breast cancer risk (La Vecchia, 1984). Benign breast disease could also at least in part, underlie the inconsistency observed in the relationship between breast cancer risk and duration, latency and recency of OC use.

Information or recall bias may also be a plausible explanation of the increased risk for short duration of use: short-term or use a long time ago might have been reported more carefully by cases than controls (Skegg, 1988). Thus, the apparently elevated risk estimates in these subgroups should be viewed with extreme caution.

The RR estimates slightly above unity for short time since first and last OC use may be explained within the framework of a multistage process of breast carcinogenesis, in terms of a late stage (promotional) effect (Day & Brown, 1980). This resembles the transient increase in the risk of breast cancer found after a full-term pregnancy (Bruzzi et al., 1988; La Vecchia et al., 1990).

These findings are not in agreement with most previous evidence referring to the pattern of risk in younger women (<40 years) (Lubin et al., 1982; Meirik et al., 1986; McPherson et al., 1987; Kay & Hannaford, 1988; Miller et al., 1989; Olsson et al., 1989; Peto, 1989; UK National Case-Control Study Group, 1989; Romieu et al., 1990; Delgado-Rodriguez et al., 1991; Weinstein et al., 1991; Rushton & Jones, 1992; Ursin et al., 1992), and in those who used OC at a younger age or before first birth (Pike et al., 1981; Meirik et al., 1986; McPherson et al., 1987). Since the confidence intervals of these estimates are wide, chance by itself is a plausible interpretation.

The possibility of bias must also be considered. Possibly the results of this study reflect the different pattern of OC use among Italian women, characterised by extremely infrequent (and probably highly selective) use in the older generations (i.e. among women above age 30 in the 1970s and 1980s) (La Vecchia et al., 1986*b*). Although the low prevalence of OC use causes major difficulties in relation to study power and possibly also to selective mechanisms, the sample size of this study, based on over 2,300 cases and 1,900 controls, provides reasonably stable risk estimates in a population whose patterns of pill use differ substantially from Northern Europe or the USA (Thomas, 1988; Romieu et al., 1990).

Another potential limitation of this study is its hospital-based design, with all the consequent implications, such as the use of hospital controls, which can be open to debate (Mantel & Haenszel, 1959). The results, however, could not be explained in terms of selection or confounding bias, since the catchment areas of cases and controls were well comparable, participation was almost complete, and allowance for several confounding factors only slightly modified the relative risk estimates. Further, the hospital setting may well improve recall of past drug use, particularly in controls, although this cannot totally eliminate a potential better recall by breast cancer cases, particularly for short term use in the distant past (Skegg, 1988).

In conclusion, the low prevalence of OC use in this Italian population seriously hampered analysis, and particularly any inference on subgroups, time factors, dose or type of preparation. Nevertheless, the large size of the dataset, the originality of the population, in terms of baseline breast cancer incidence and patterns of hormone use (La Vecchia et al., 1986*b*), and the consistency of its results with the overall evidence from other studies (Mann, 1990) add useful information to a debate of major public health relevance. The indications emerging from this study for the use of OC and the risk of breast cancer are reassuring and should help in assessing the pattern of breast cancer risk better for various time-related factors of OC use in a Southern European population.

This work was conducted within the framework of the National Research Council (CNR) Applied Projects 'Clinical Application of Oncological Research' (contracts No. 92.02384.PS39 and No. 9202324.PS39), and 'Prevention and Control of Disease Factors' (contract No. 91.00285.PF41), and with the contribution of the Italian Association for Cancer Research and the Italian League against Tumors, Milan and Mrs A. Marchegiano Borgomainerio. The Authors thank Mrs J. Baggott, Miss M.P. Bonifacino and the G.A. Pfeiffer Memorial Library staff for editorial assistance.

### References

BRESLOW, N.E. & DAY, N.E. (1980). Statistical methods in cancer research. Vol. 1. The analysis of case-control studies. *IARC Sci. Publ.*, 32.

BRINTON, L.A., HOOVER, R., SZKLO, M. & FRAUMENI, J.F. Jr (1982). Oral contraceptives and breast cancer. *Int. J. Epidemiol.*, **11**, 316–322.

BRUZZI, P., NEGRI, E., LA VECCHIA, C., DECARLI, A., PALLI, D., PARAZZINI, F. & ROSSELLI DEL TURCO, M. (1988). Short-term increase in risk of breast cancer after full term pregnancy. *BMJ*, **297**, 1096–1098.

DAY, N.E. & BROWN, C.C. (1980). Multistage models and primary prevention of cancer. *J. Natl Cancer Inst.*, **64**, 977–989.

DELGADO-RODRIGUEZ, M., SILLERO-ARENAS, M., RODRIGUEZ-CONTRERAS, R.L., GIGOSOS, R. & GLAVEZ VARGAS, R. (1991). Oral contraceptives and breast cancer. A meta-analysis. *Rev. Epidemiol. Sante Publ.*, **39**, 165–181.

DOLL, R. (1990). What conclusions can we reach and how do we best assist women to make informed choices regarding their use of oral contraceptives. In *Oral Contraceptives and Breast Cancer*, Mann, R.D. (ed.) pp. 395–400. Parthenon Publ. Carnforth (UK).

EWERTZ, M. (1992). Oral contraceptives and breast cancer risk in Denmark. *Eur. J. Cancer*, **28A**, 1176–1181.

KAY, C.R. & HANNAFORD, P.C. (1988). Breast cancer and the pill. A further report from the Royal College of General Practitioners' oral contraception study. *Br. J. Cancer*, **58**, 675–680.

LA VECCHIA, C. (1984). Benign breast disease, oral contraceptive use, and the risk of breast cancer. *J. Chronic Dis.*, **37**, 869–870.

LA VECCHIA, C. (1992). Oral contraceptives and breast cancer. *The Breast*, **2**, 76–81.

LA VECCHIA, C., BRUZZI, P. & BOYLE, P. (1990). Some further consideration on the role of oral contraceptives in breast carcinogenesis. *Tumori*, **76**, 220–224.

LA VECCHIA, C., DECARLI, A., FASOLI, M., FRANCESCHI, S., GENTILE, A., NEGRI, E., PARAZZINI, F. & TOGNONI, G. (1986*a*). Oral contraceptives and cancers of the breast and of the female genital tract. Interim results from a case-control study. *Br. J. Cancer*, **54**, 311–317.

LA VECCHIA, C., DECARLI, A., PARAZZINI, F., GENTILE, A., NEGRI, E., & FRANCESCHI, S. (1986*b*). Determinants of oral contraceptive use in Northern Italy. *Contraception*, **34**, 145–156.

LA VECCHIA, C., PARAZZINI, F., NEGRI, E., BOYLE, P., GENTILE, A., DECARLI, A. & FRANCESCHI, S. (1989). Breast cancer and combined oral contraceptives: an Italian case-control study. *Eur. J. Cancer Clin. Oncol.*, **25**, 1613–1618.

LUBIN, J.H., BURNS, P.E., BLOT, W.J., LEES, A.W., MAY, C., MORRIS, L.E. & FRAUMENI, J.F. Jr (1982). Risk factors for breast cancer in women in Northern Alberta, Canada, as related to age at diagnosis. *J. Natl Cancer Inst.*, **68**, 211–217.

MANN, R.D. (1990). Oral contraceptives and breast cancer risk. A problem of consent. In *Oral Contraceptives and Breast Cancer*, Mann, R.D. (ed.) pp. 7–33. Parthenon Publ. Carnforth (UK).

MANTEL, N. & HAENSZEL, W. (1959). Statistical aspects of the analysis of data from retrospective studies of disease. *J. Natl Cancer Inst.*, **22**, 719–748.

MCPHERSON, K., VESSEY, M.P., NEIL, A., DOLL, R., JONES, L. & ROBERTS, M. (1987). Early oral contraceptive use and breast cancer: results of another case-control study. *Br. J. Cancer*, **56**, 653–660.

MEIRIK, O., LUND, E., ADAMI, H.-O., BERGSTROM, R., CHRISTOFFERSEN, T. & BERGSJO, P. (1986). Oral contraceptive use and breast cancer in young women. A joint national case-control study in Sweden and Norway. *Lancet*, **2**, 650–654.

MILLER, D.R., ROSENBERG, L., KAUFMAN, D.W., STOLLEY, P., WARSHAUER, M.E. & SHAPIRO, S. (1989). Breast cancer before age 45 and oral contraceptive use: new findings. *Am. J. Epidemiol.*, **129**, 269–280.

OLSSON, H. (1989). Oral contraceptives and breast cancer. A review. *Acta Oncol.*, **28**, 849–863.

OLSSON, H., MOLLER, T.R. & RANSTAM, J. (1989). Early oral contraceptive use and breast cancer among premenopausal women: final report from a study in southern Sweden. *J. Natl Cancer Inst.*, **81**, 1000–1004.

PAUL, C., SKEGG, D.C.G., SPEARS, G.F.S. & KALDOR, J.M. (1986). Oral contraceptives and breast cancer: a national study. *BMJ*, **293**, 723–726.

PETO, J. (1989). Oral contraceptives and breast cancer: is the CASH study really negative? *Lancet*, **1**, 552.

PIKE, M.C., HENDERSON, B.E., CASAGRANDE, J.T., ROSARIO, I. & GRAY, G.E. (1981). Oral contraceptive use and early abortion as risk factors for breast cancer in young women. *Br. J. Cancer*, **43**, 72–76.

PRENTICE, R.L. & THOMAS, D.B. (1987). On the epidemiology of oral contraceptives and disease. *Adv. Cancer Res.*, **49**, 285–401.

ROMIEU, I., BERLIN, J.A. & COLDITZ, G. (1990). Oral contraceptives and breast cancer. Review and meta-analysis. *Cancer*, **66**, 2253–2263.

ROSENBERG, L., MILLER, D.R., KAUFMAN, D.W. & HELMRICH, S.P., STOLLEY, P.D., SCHOTTENFELD, D. & SHAPIRO, S. (1984). Breast cancer and oral contraceptive use. *Am. J. Epidemiol.*, **119**, 167–176.

RUSHTON, L. & JONES, D.R. (1992). Oral contraceptives use and breast cancer risk: a meta-analysis of variations with age at diagnosis, parity and total duration of oral contraceptive use. *Br. J. Obstet. Gynaecol.*, **99**, 239–246.

SCHLESSELMAN, J.J., STADEL, B.V., MURRAY, P. & LAI, S. (1988). Breast cancer in relation to early use of oral contraceptives. No evidence of a latent effect. *JAMA*, **259**, 1828–1833.

SKEGG, D.C.G. (1988). Potential for bias in case-control studies of oral contraceptives and breast cancer. *Am. J. Epidemiol.*, **127**, 205–212.

STADEL, B.V., RUBIN, G.L., WEBSTER, L.A., SCHLESSELMAN, J.J. & WINGO, P.A. (1985). Oral contraceptives and breast cancer in young women. *Lancet*, **2**, 970–973.

THOMAS, D.B. (1988). The breast. In *Symposium on Improving Safety Requirements for Contraceptive Steroids*. World Health Organization: Geneva.

UK NATIONAL CASE-CONTROL STUDY GROUP (1989). Oral contraceptive use and breast cancer risk in young women. *Lancet*, **1**, 974–982.

URSIN, G., ARAGAKI, C.C., PAGANINI-HILL, A., SIEMIATYCKI, J., THOMPSON, W.D. & HAILE, R.W. (1992). Oral contraceptives and premenopausal bilateral breast cancer: a case-control study. *Epidemiology*, **3**, 414–419.

VESSEY, M., BARON, J., DOLL, R., MCPHERSON, K. & YEATES, D. (1982). Oral contraceptives and breast cancer: final report of an epidemiological study. *Br. J. Cancer*, **47**, 455–462.

VESSEY, M.P., MCPHERSON, K., VILLARD-MACKINTOSH, L. & YEATES, D. (1989). Oral contraceptives and breast cancer: latest findings in a large cohort study. *Br. J. Cancer*, **59**, 613–617.

WEINSTEIN, A.L., MAHONEY, M.C., NASCA, P.C., LESKE, M.C. & VARMA, A.O. (1991). Breast cancer risk and oral contraceptive use: results from a large case-control study. *Epidemiology*, **2**, 353–358.